# Optimizing Soil Health and Sorghum Productivity through Crop Rotation with Quinoa

**DOI:** 10.3390/life14060745

**Published:** 2024-06-12

**Authors:** Guang Li, Aixia Ren, Sumera Anwar, Lijuan Shi, Wenbin Bai, Yali Zhang, Zhiqiang Gao

**Affiliations:** 1 College of Agriculture, Shanxi Agricultural University, Jinzhong 030800, China; lghebe@126.com (G.L.); rax_renaixia@163.com (A.R.); shiliuan_gls@163.com (L.S.); jzmujin@163.com (Y.Z.); 2 Department of Botany, Government College Women University Faisalabad, Faisalabad 38000, Pakistan; sumeraanwar@mail.hzau.edu.cn

**Keywords:** *Sorghum bicolor* (L.) Moench, *Chenopodium quinoa* Willd., cropping methods, soil properties, continuous cropping, yield

## Abstract

Crop rotation has been considered a potential solution to mitigate the negative effects of the continuous cropping of sorghum, including soil quality issues, inadequate plant development, and diminished yield and quality. A two-year field experiment was conducted to compare the effects of sorghum–sorghum continuous cropping and quinoa–sorghum rotation on soil properties and sorghum yield. The treatments were arranged in a randomized complete block design with three replicates. Sorghum seeds (Jinza 22) and quinoa seeds (‘Jiaqi 1’ variety) were used. Soil samples were collected before and during the experiment for the analysis of physicochemical properties. The yield traits of sorghum were measured at maturity. The results showed that soil nutrients and organic matter were higher in the top 0–20 cm soil depth compared to 20–40 cm depth, with significant differences observed between cropping systems. Sorghum–quinoa cropping increased soil total N and organic matter, particularly at the jointing and maturity stages of sorghum. However, the available phosphorus was higher under continuous cropping at all growth stages. Crop rotation significantly improved sorghum yield traits, including spike fresh weight, spike dry weight, grain weight per spike, and grain yield per hectare. A correlation analysis revealed positive relationships between soil total N, organic matter, and sorghum yield. Overall, sorghum–quinoa rotation demonstrated potential for improving soil fertility and enhancing crop productivity compared to continuous cropping, although further studies are needed to explore the long-term effects and optimize management practices.

## 1. Introduction

The cropping system, or cropping sequence, is a simple practice with a significant impact on agroecological conditions [1,2]. Plants grown under different cropping systems interact differently with the environment, which ultimately determines yield, growth, nutrient uptake, and soil health [3]. With the advancement of mechanized agriculture, continuous cropping has become a prominent method for growing major crops. Monocropping, the continuous cultivation of a single crop over the years, is preferred as it allows farmers to utilize machinery efficiently, enhancing the ease of planting and harvesting [4].

Despite the role of monocropping in supporting a substantial portion of the global food supply, monoculture is contentious in modern agriculture [5]. Continuous cropping depletes soil nutrients, reduces organic carbon content and microbial diversity, and alters soil properties [6]. These changes result in decreased yield and quality [7,8]. Consequently, this method requires high fertilizer input, leading to nitrogen leaching into groundwater and soil erosion. Additionally, it increases the risk of weeds, pests, and disease outbreaks, necessitating high herbicide and pesticide input.

Crop rotation is a multiple cropping method that resembles a semi-natural habitat. It involves cultivating two or more crops in succession. This practice is considered beneficial for enhancing landscape complexity and diversity [9]. Crop rotation requires fewer resources, enhances the quality of the agroecosystem, increases microbial diversity [10], and improves soil fertility and soil structure [11,12]. The primary goal of crop rotation is to increase diversity, reduce soil erosion by providing a cover crop, and enhance yield per unit area by optimizing resource use [13].

Crop rotation increases productivity and yield stability by optimizing space and time use compared to monocropping [14]. It creates a more favorable soil environment for subsequent crops through changes in the soil properties, nutrient availability [15], soil pH, and soil microbial diversity [16], while reducing weeds and pest infestations [17]. These factors collectively contribute to higher and more stable yields under diverse environmental conditions [18]. Additionally, crop rotation can reduce pest infestations, making pest control more manageable [17,19]. Intercropping and crop rotations are also effective in controlling weed density, reducing the need for herbicides, and minimizing competition with weeds [19,20]. Furthermore, mixing deep and shallow-rooted crops increases N uptake and reduces N losses [21]. However, the improved root function may also lead to competition for water and nutrients, with dominant crops like cereals having an advantage over the less dominant ones [22].

Sorghum (*Sorghum bicolor* (L.) Moench) has been cultivated in China for a very long time, almost 40–50 centuries. This cereal crop is valued for its multiple uses in food, feed, and bioethanol production [23,24]. Although sorghum is a minor crop in China, it can be grown on 0.24% of marginal land due to its drought resistance and utilization of energy [25]. However, the challenges in sorghum cultivation as a sole crop include soil quality issues [26,27], inadequate plant development, increased susceptibility to disease [28], and reduced yield and quality [14].

Crop rotation has been suggested as a potential solution to mitigate the negative impacts associated with continuous cropping practices [7]. Simão et al. [14] reported that 44 years of sorghum crop rotation with soybean significantly enhanced sorghum yield compared to continuous sorghum. Similarly, Malobane et al. [12] reported that sorghum–vetch rotation increased soil nitrate, ammonium, and phosphorus content compared to continuous sorghum cropping. Five-year sorghum crop rotations with soybean and cotton in clayey soil increased the sorghum yield by 840–1068 kg ha^−1^ and 898–1047 kg ha^−1^, respectively, compared to sorghum monocropping [29]. Soybean, with its low carbon ratio and higher turnover rates, contributes less to soil organic content under continuous cropping. In contrast, rotating sorghum with other crops maintains higher soil organic content and water-holding capacity due to greater above-ground biomass, higher carbon ratios, and more fibrous roots [30].

Quinoa (*Chenopodium quinoa* Willd.) is an under-utilized crop that has been grown for hundreds of years, although it has recently gained popularity due to its nutritional value. Furthermore, quinoa is highly tolerant to pests, arid conditions, and saline and nutrient-deficient soil [31]. Moreover, its extensive root system (>1.2 m depth) reduces water evaporation and makes it adaptable to various ecological conditions, including semi-arid and arid soils [32]. Quinoa has been shown to improve forage yield [33], millet yield [34], and potato yield [20] when used in intercropping and crop rotation.

However, crop rotations do not always result in increased yield, as outcomes depend on factors such as allelopathic effects, crop compatibility, soil nutrient status, and water availability. For instance, quinoa rotation reduced wheat and chickpea yield in dry Mediterranean areas [35], and sorghum–maize crop rotation was not recommended in sandy soil where maize yield reduction was observed [15]. Therefore, different cropping systems should be tested under diverse growth conditions.

The pressures of continuous cropping can be alleviated by implementing sorghum–quinoa rotations, thereby enhancing sorghum yield. A deep understanding of the ecosystem is necessary to improve soil quality and yield efficiency through such rotations. Based on previous findings, we hypothesized that sorghum–quinoa rotation can improve soil quality by affecting N content and availability, organic matter content, and pH, thereby influencing sorghum yield. To test the hypothesis, we designed a preliminary field experiment to compare sorghum continuous cropping and quinoa–sorghum rotation. This study systematically examines hydrolysable N content, total N content, available K content, organic matter, and pH at different soil depths and growth stages, along with sorghum yield. By understanding the interactions between soil properties and yield, this project aims to contribute to agricultural sustainability and resilience in the region.

## 2. Materials and Methods

### 2.1. Site and Experiment Design

**Figure 1:**
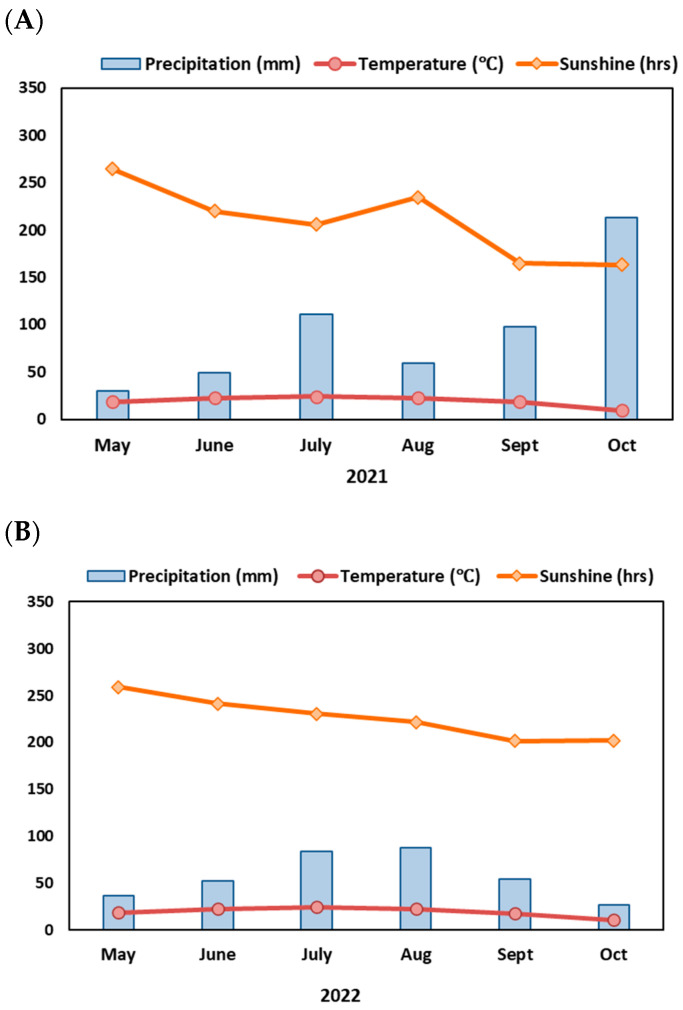
The average monthly temperature, total monthly precipitation, and accumulated sunlight hours during the sorghum and quinoa crop growth period in (**A**) 2021 and (**B**) 2022.

The two-year trial began in May 2021 at the Dongbai Experimental Base of the Sorghum Research Institute, Shanxi Agricultural University, China. The experiment consisted of two cropping methods as treatments: sorghum–fallow–sorghum continuous cropping and quinoa–sorghum rotation. The sorghum seeds (Jinza 22) utilized in this study were sourced from the Sorghum Research Institute, Academy of Agriculture Sciences, Taiyuan, Shanxi Province, China. The seeds of the ‘Jiaqi 1’ variety of quinoa were procured from Shanxi Jiaqi Quinoa Development Co., Ltd. (Shuozhou, China). The meteorological data for the site are given in Figure 1.

### 2.2. Field Preparation

The compound fertilizer (N:P:K 20:20:5) was applied at a rate of 750 kg ha^−1^ and mixed in the soil with rotary tillage at a depth of 15 cm. The seeds of both crops were sown with a drill on 5 May 2021 and 2 May 2022. The area under each cropping system was 100 m^2^ (5 m × 20 m), and each treatment was repeated three times. The sorghum was planted at a planting density of 117,000 plants ha^−1^, a row spacing of 50 cm, and a plant spacing of 17 cm, and the quinoa crop was planted at a planting density of 135,000 plants ha^−1^, a row spacing of 50 cm, and a plant spacing of 15 cm. The crops were harvested on 12 October 2021, and 9 October 2022. Under sorghum–sorghum, sorghum was sown in both years (2021 and 2022), while under the sorghum–quinoa rotation, quinoa was sown in 2021 and sorghum was sown in 2022. In the first year (2021), the soil properties were estimated at two different depths under two cropping systems. In the second year (2022), the soil properties were determined at different growth stages of sorghum, and sorghum yield was determined. The treatment design in both years was a randomized complete block design with three blocks.

The soil of the field area was brown and calcareous. The physicochemical properties of soil are given in Table 1.

### 2.3. Soil Analysis

Soil samples were collected before starting the experiment in April 2021 from a depth of 200 cm using a core. Then, during the experiment in 2021, soil samples were collected at the maturity stage of sorghum on 12 October from 0 to 20 and 20 to 40 cm soil depth. In 2022, soil samples were collected at the jointing stage on 1 June, the heading stage on 5 August, and the maturity stages on 9 October of sorghum. From each replicate plot, three soil samples were collected, which were oven-dried, mixed, and sieved through a 2 mm sieve.

The soil pH of soil water suspension (1:2.5 *v*/*w*) was standardized by the potentiometric method [36]. Soil total nitrogen was quantified by the potassium dichromate–sulfuric acid digestion–distillation method [37]. Soil alkali-hydrolyzable nitrogen was quantified by the alkaline diffusion method [38].

Available phosphorus was quantified by the sodium bicarbonate extraction-molybdenum–antimony colorimetric method [39]. Available potassium was carried out by the ammonium acetate extraction–flame photometry method [40]. Cation exchange capacity was determined by the ammonium acetate method [40].

For the soil organic content, the Walkley and Black method [41] was employed. For this, air-dried soil (0.5 g) was mixed with 5 mL potassium dichromate (1 mol L^−1^) and 5 mL sulfuric acid and digested by heating at 100 °C for 35 min. The remaining chromate was determined spectrophotometrically at 600 nm.

### 2.4. Measurement of Yield Traits

In 2022, at the maturity stage of sorghum, two rows were harvested from each subplot (block), and panicle fresh weight was recorded. Then, the panicles were dried in an oven, and the dry weight was measured. After this, the grains were removed, and the weight of grains per spike was weighed. The spike fertility index, or spike harvest index, was calculated as a ratio of the grain weight of the spike to the spike dry weight [42]. Then, the rest of the panicles were harvested from the field and sun-dried until the moisture content became 14% or less, and the grain yield (kg m^−2^) was calculated and converted to tons per hectare.

### 2.5. Statistical Analysis

The soil and yield data recorded during the two years were analyzed by ANOVA, and differences among treatments were determined by a post hoc test (Tukey test at *p* < 0.05). The correlation graph of soil traits at the maturity stage in 2022 and yield traits was drawn by the Corrplot package of RStudio (version 2023.06.1+524).

## 3. Results

### 3.1. Soil Properties at Different Soil Depths under Continuous Cropping and Crop Rotation

**Figure 2:**
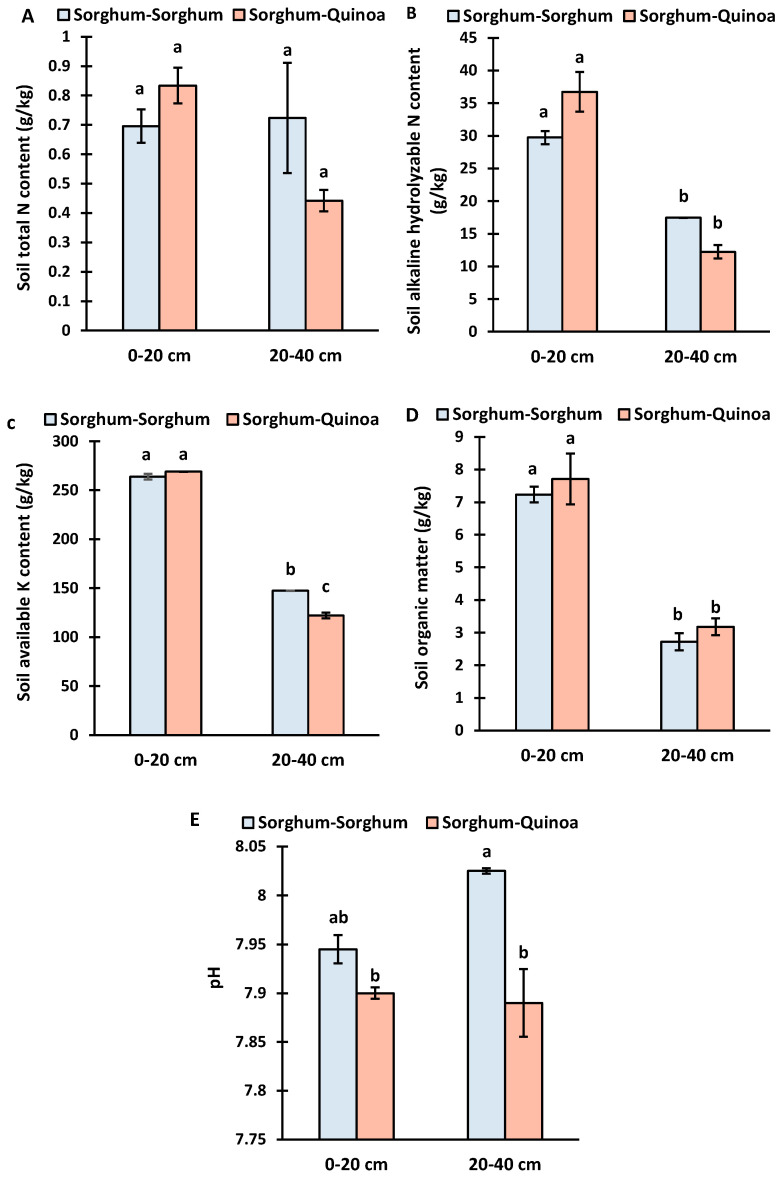
Soil physio-chemical properties from 0 to 20 and 20 to 40 cm soil depth under sorghum continuous cropping and sorghum–quinoa crop rotation in 2021. (**A**) soil total nitrogen content, (**B**) soil alkaline hydrolysable nitrogen content, (**C**) soil available potassium content, (**D**) soil organic matter, (**E**) soil pH. Different letters on bars indicate significant differences according to the Tukey test (*p* < 0.05). Vertical bars correspond to the standard error.

The total N content, alkaline hydrolyzable content, available K content, organic matter, and pH were measured from 0 to 20 and 20 to 40 cm soil depths at the maturity stage of sorghum in 2021 under sole cropping and crop rotation with quinoa. The soil total N content was not significantly affected by the soil depth and cropping method (Figure 2A; Table S1).

The alkaline hydrolyzable content (Figure 2B), available K content (Figure 2C), and organic matter content (Figure 2D) in 0–20 depth were significantly higher than in 20–40 cm soil depth, although alkaline hydrolyzable N and soil organic matter were not affected by cropping methods in 2021 (Table S1).

The pH at the start of the experiment was 8.98 (Table 1) and was decreased under both cropping systems during experimentation (Figure 2E). The pH under crop rotation was lower than under continuous cropping. The pH value was not affected by the soil depth and the interaction of the cropping system and soil depth (Table S1). Overall, the soil nutrients and organic matter content were highest under the sorghum–quinoa rotation in 0–20 cm soil depth, but mostly, the effect of crop rotation was non-significant during the first year of the crop rotation.

### 3.2. Soil Properties at Different Growth Stages of Sorghum Continuous Cropping and Crop Rotation

In the following year, the soil properties from 0 to 20 cm soil depths were studied at the jointing, heading, and maturity stages of sorghum grown as continuous cropping and crop rotation with quinoa. The total N, alkaline hydrolysable N, available K, and organic matter content were significantly affected by the cropping system (Table S2).

**Figure 3:**
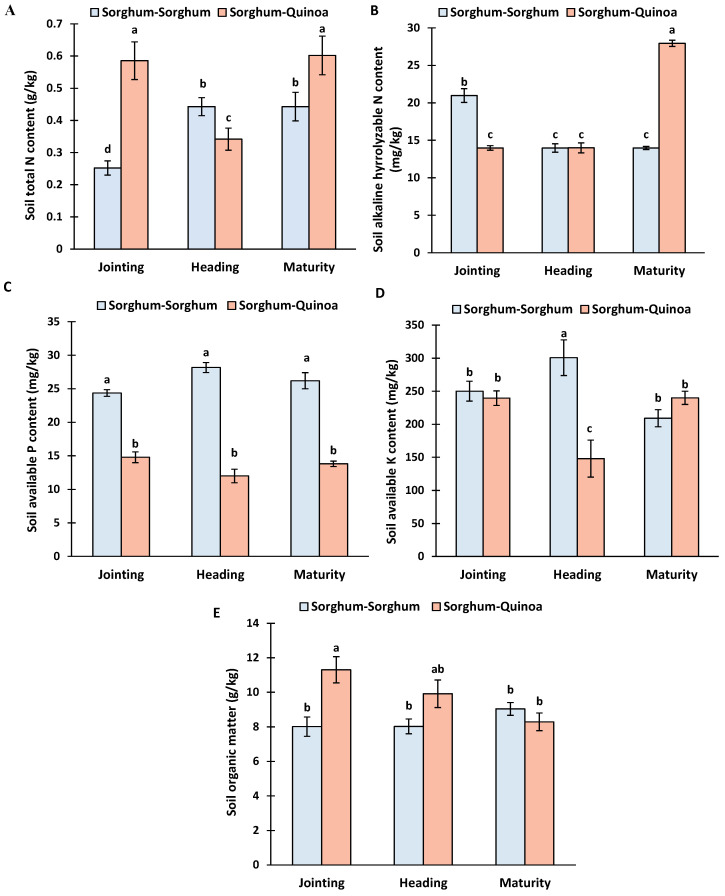
Soil chemical characteristics under sorghum continuous cropping and sorghum–quinoa crop rotation in 2022. (**A**) soil total nitrogen content, (**B**) soil alkaline hydrolyzable nitrogen content, (**C**) soil available phosphorous content, (**D**) soil available potassium content, (**E**) soil organic matter. Different letters on bars indicate significant differences according to the Tukey test (*p* < 0.05). Vertical bars correspond to the standard error.

The total N, alkaline hydrolysable N, and available K were significantly affected by the growth stages and the interaction between the cropping system and growth stages. The sorghum–quinoa cropping showed higher soil total N contents at the jointing and maturity stages compared to sorghum continuous cropping, while at the heading stage, there was an opposite trend (Figure 3A).

The alkaline hydrolyzable N content at the jointing stage was higher in sorghum continuous cropping than in crop rotation, while the heading stage was similar in both cropping systems and then increased under crop rotation at the maturity stage of sorghum (Figure 3B).

Soil available P was higher under sorghum continuous cropping than under sorghum–quinoa cropping during all three growth stages (Figure 3C). The soil available K content under the heading was higher under continuous cropping, and at maturity, it was higher under crop rotation (Figure 3D). Soil organic matter at the jointing stage was higher under crop rotation than single cropping, while it was similar in both cropping methods under heading and maturity (Figure 3E).

### 3.3. Yield of Sorghum under Sorghum Continuous Cropping and Crop Rotation with Quinoa

The yield traits of sorghum were calculated in the second year of the cropping treatment. The spike fresh weight, spike dry weight, grain weight per spike, and grain yield per hectare were significantly higher under the sorghum–quinoa rotation than in the continuous cropping of sorghum (Table S2).

**Figure 4:**
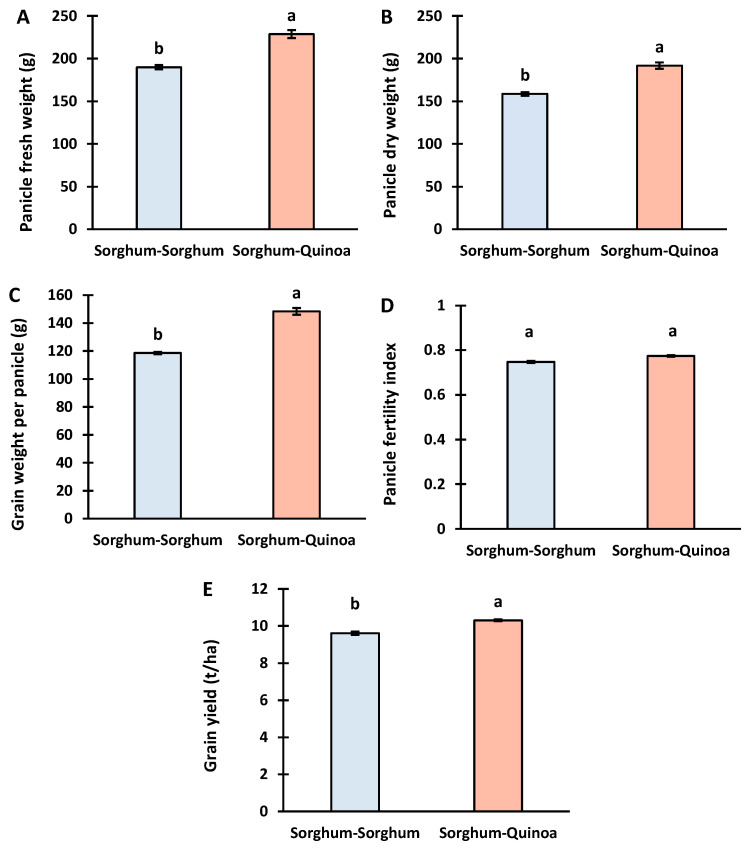
The yield traits of sorghum under sorghum continuous cropping and sorghum–quinoa crop rotation in 2022. (**A**) panicle fresh weight, (**B**) panicle dry weight, (**C**) grain weight per panicle, (**D**) panicle fertility index, (**E**) grain yield. Different letters on bars indicate significant differences according to the Tukey test (*p* < 0.05). Vertical bars correspond to the standard error.

These results showed the beneficial effect of 2 years of sorghum–quinoa rotation on sorghum yield. Sorghum–quinoa rotation increases the spike fresh weight (Figure 4A) and spike dry weight (Figure 4B) by 20.5 and 20% compared to sorghum continuous cropping. The 2 years of crop rotation increased the grain weight per spike (Figure 4C) and grain yield per hectare (Figure 4D) of sorghum by 25 and 7.3% compared to mono-cropping.

### 3.4. Correlation of Soil Nutrients and Sorghum Yield Traits

**Figure 5:**
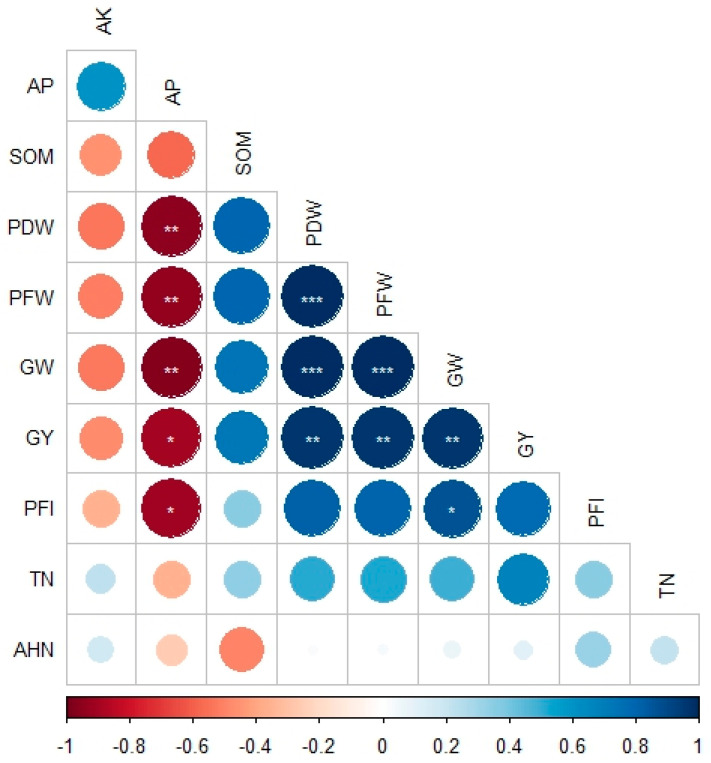
Correlation of soil properties and yield traits of sorghum. TN: total nitrogen content in soil; AHN: alkaline hydrolyzable nitrogen content in soil; AP: available phosphorus content in soil; SOM: soil organic matter; PDW: panicle dry weight; PFW: panicle fresh weight; GW: grain weight per panicle; PFI: panicle fertility index; GY: grain yield per area. *, **, and *** indicate significance at 0.05, 0.01, and 0.001 probability levels.

Grain yield was found to be positively related to the total N and organic matter content in soil (Figure 5), while the available P was negatively related. Furthermore, grain yield was positively related to panicle weight, grain weight, and panicle fertility index.

## 4. Discussion

### 4.1. Soil Properties at Different Soil Depths under Continuous Cropping and Crop Rotation

In this study, we investigated the impact of sorghum crop rotation with quinoa on soil properties and sorghum yield. The soil properties were assessed before the commencement of the experiment and after the first and second years of cropping systems. Soil properties were measured at two different depths (0–20 cm and 20–40 cm) at the maturity stage of sorghum and quinoa crops in 2021. The total nitrogen content did not show significant variation with soil depth or cropping method (Figure 2A; Table S1). This finding suggests that the total N content remains relatively stable regardless of the cropping practice or soil depth within the first 40 cm of soil.

In contrast, the alkaline hydrolyzable N, available K content, and soil organic matter contents were significantly higher in the 0–20 cm soil depth compared to the 20–40 cm depth (Figure 2B–D). This pattern aligns with typical soil nutrient distribution patterns [43], likely due to the input of fertilizers, the addition of plant matter (litter cover), higher microbial activity, and root distribution in the upper soil layer, which enhances nutrient availability and organic matter decomposition. Despite these differences, alkaline hydrolyzable N and soil organic matter were not significantly affected by the cropping method in the first year (Table S1).

The comparatively lower nutrient levels in the 20–40 cm soil depth following crop rotation may suggest increased nutrient uptake in this soil depth by the quinoa crop. However, the non-significant difference observed between cropping systems may imply that the effects of cropping systems on soil properties develop gradually over multiple cropping cycles [44,45]. Previous research indicates that crop rotation has the potential to increase or maintain soil fertility. Although the increase in nutrient content in the upper soil layer indicates the potential of crop rotation to improve soil fertility [45,46].

The soil pH, initially at 8.98 (Table 1), decreased under both monocropping and crop rotation. A more pronounced decrease in pH was observed under sorghum rotation with quinoa compared to continuous cropping (Figure 2E) and in the deeper soil depth (20–40 cm) under both cropping systems. This suggests that crop rotation may have a greater impact on soil pH compared to continuous cropping. The reduction in pH under crop rotation could be attributed to increased organic matter decomposition, higher microbial activity, and root exudation to enhance nutrient availability [44,47].

### 4.2. Soil Properties at Different Growth Stages under Continuous Cropping and Crop Rotation

In the following year, we evaluated the soil properties at different growth stages of sorghum (jointing, heading, and maturity) under both cropping systems. Our results revealed variations in the total N, alkaline hydrolyzable N, available P, available K, and organic matter between continuous cropping and crop rotation, as well as across different growth stages (Figure 3; Table S2). These results indicate that crop rotation with quinoa positively influences soil fertility and nutrient dynamics over time.

The total N, alkaline hydrolyzable N, and available K contents were significantly affected by the growth stages and the interaction between the cropping system and growth stages. Specifically, the sorghum–quinoa rotation showed higher total N content at the jointing and maturity stages compared to continuous cropping, while an opposite trend was observed at the heading stage (Figure 3A). The soil’s alkaline hydrolyzable nitrogen is used as an indicator of nitrogen mineralization potential [48]. The soil alkaline hydrolyzable nitrogen was highest at the maturity stage in 2022 after two years of crop rotation. This suggests that crop rotation benefits N mineralization and availability during critical growth stages, potentially enhancing sorghum growth and yield.

The soil organic matter was higher under crop rotation at the jointing and heading stages (Figure 3E). These results showed the beneficial effect of crop rotation on the total nitrogen and organic matter in 0–20 cm soil. The changes in soil properties reflect a complex relationship between plant roots, rhizospheric microbes, and microbial activities. Plant roots excrete organic acids in soil, which attract microbes, and in turn, microbial enzymes such as urease increase soil nitrogen [49].

Similarly, the soil available P content was higher under continuous cropping at all growth stages (Figure 3C), whereas the soil available K content varied between continuous cropping and crop rotation across different growth stages (Figure 3D). Similarly, Wang et al. [45] reported a decline in the Olsen phosphorous content after 9 to 10 cycles of various crop rotations. The low P at all stages and low N at the heading stage might be linked to the high P removal as a result of crop rotation as compared to continuous cropping, as observed by many researchers [45,50].

### 4.3. Yield of Sorghum under Continuous Cropping and Crop Rotation with Quinoa

In the second year of cropping treatment, a significant enhancement in sorghum yield traits was observed under the sorghum–quinoa rotation compared to continuous cropping (Figure 4). These results suggest the beneficial effects of crop rotation on sorghum yield. Crop rotation led to a significant increase in panicle dry weight, grain weight per panicle, and grain yield per hectare compared to continuous cropping, indicating improved productivity under crop rotation conditions. Similar increases in maize yield and forage quality were observed by Koca [33] through crop rotation with 25, 50, and 75% of quinoa crops.

The sorghum–quinoa rotation increased the fresh and dry weight of the panicle by 20.5 and 20% compared to sorghum continuous cropping. Furthermore, the grain weight per panicle increased by 25%. The higher panicle weight contributes to the yield increase as it indicates high photosynthate allocation. However, increasing the panicle weight does not necessarily result in a proportional increase in the grain yield per panicle. If the grains are produced through panicle photosynthesis, having more panicles per plant could lead to increased shading between the panicles. As a result, there would be a greater investment in panicle mass for only a slightly higher seed output. To sort this out, the panicle fertility index of sorghum was calculated as a ratio of seed weight panicle^−1^ to panicle weight. In a competitive environment, this ratio is more useful to identify superior yield traits, as higher competition decreases panicle fitness [51]. The panicle fertility index of sorghum increased from 0.77 under crop rotation to 0.74 under continuous cropping. However, the non-significant effect indicates no difference between the cropping systems in resource allocation to grains with respective panicles. The grain yield per hectare of sorghum was 7.3% higher under sorghum–quinoa rotation than under monocropping.

Overall, soil fertility, encompassing physical, chemical, and biological aspects, is crucial for crop yields in agricultural ecosystems [46]. It refers to a soil’s ability to support plant productivity and improve nutrient quality. Our correlation analysis revealed important relationships between soil nutrients and sorghum yield traits (Figure 5). Grain yield was positively correlated with total N and organic matter content in the soil, indicating the importance of these nutrients for crop productivity. Furthermore, the higher hydrolyzable nitrogen and organic matter in soil result in higher nutrient uptake by plants, increasing productivity [45]. The mineralizable N, organic matter, and inorganic N content in soil are important indicators of soil health. Agomoh et al. [52] reported that the changes in these indicators accounted for a 34% variation in soybean yield under crop rotation. Conversely, available P showed a negative correlation with grain yield, suggesting potential limitations imposed by phosphorus availability.

## 5. Conclusions

Sorghum–quinoa rotation demonstrated improvements in soil fertility traits and enhanced crop productivity compared to continuous cropping. Soil total nitrogen and organic matter were higher under crop rotation, particularly at specific growth stages of sorghum. Although available phosphorus was higher under continuous cropping, the overall soil health indicators favored crop rotation. Sorghum–quinoa rotation increased the panicle fresh weight, panicle dry weight, grain weight per panicle, and grain yield per hectare by 20.5%, 20%, 25%, and 7.3%, respectively, compared to mono-cropping. Correlation analysis further confirmed the positive relationship of soil total nitrogen and organic matter with sorghum yield. These findings suggest that sorghum–quinoa rotation has the potential to improve soil nutrient dynamics and enhance crop productivity compared to continuous cropping, although the specific effects may vary depending on factors such as soil depth and crop growth stage. Further, long-term research is needed to fully explore the effects of crop rotations and optimize other management practices on soil and crop performance.

## Figures and Tables

**Table 1:** Initial soil properties before initiation of the experiment.

Soil Traits	Value
pH	8.98
Total salt content (%)	0.042
Available phosphorus (mg kg^−1^)	8.7
Available potassium (mg kg^−1^)	159
Organic matter (g kg^−1^)	6.66
Total nitrogen (g kg^−1^)	0.511
Total potassium (g kg^−1^)	19.6
Total phosphorous (g kg^−1^)	0.68
Cation exchange capacity (cmol kg^−1^)	18.9
Alkalinity (%)	13.6
Mineral nitrogen (mg kg^−1^)	35.2
Soluble carbon (g kg^−1^)	0.175

## Data Availability

The original contributions presented in the study are included in the article/Supplementary Material, further inquiries can be directed to the corresponding author.

## Supplementary Materials

The following supporting information can be downloaded at https://www.mdpi.com/2075-1729/14/6/745/s1, Table S1: Analysis of variance (*p*-values) for the soil traits under two cropping systems and soil depths in 2021; Table S2: Analysis of variance (*p*-values) for the soil properties under two cropping systems and growth stages of sorghum, and yield traits under two cropping systems in 2022.
