# Correction: Li et al. Optimizing Soil Health and Sorghum Productivity through Crop Rotation with Quinoa. *Life* 2024, *14*, 745

**DOI:** 10.3390/life14070866

**Published:** 2024-07-11

**Authors:** Guang Li, Aixia Ren, Sumera Anwar, Lijuan Shi, Wenbin Bai, Yali Zhang, Zhiqiang Gao

**Affiliations:** 1 College of Agriculture, Shanxi Agricultural University, Jinzhong 030800, China; lghebe@126.com (G.L.); rax_renaixia@163.com (A.R.); shiliuan_gls@163.com (L.S.); jzmujin@163.com (Y.Z.); 2 Department of Botany, Government College Women University Faisalabad, Faisalabad 38000, Pakistan; sumeraanwar@mail.hzau.edu.cn

## Addition of a Corresponding Author

The author Wenbin Bai has been changed to the second corresponding author. The corrected Author Contributions statement appears here. The authors state that the scientific conclusions are unaffected. This correction was approved by the Academic Editor. The original publication [1] has also been updated.
