# Supplementary material for: Optimizing Soil Health and Sorghum Productivity through Crop Rotation with Quinoa
Source: Life (Basel). 2024 Jun 12;14(6):745. doi: 10.3390/life14060745 (PMC11205141; doi:10.3390/life14060745)
Supplement: Supplementary file 1 [file life-14-00745-s001.zip › life-3019296-supplementary.pdf]

Table S1 Analysis of variance (*P*-values) for the soil traits under two cropping systems and soil depths in 2021

| Source of variation | Soil total N content | Soil alkaline hydrolyzable N | Soil available K content | Soil organic matter | pH |
|---|---|---|---|---|---|
| Cropping system | 0.543ns | 0.630ns | 0.0009*** | 0.125ns | 0.004* |
| Soil depth | 0.155ns | 0.000*** | 0.000*** | 0.000*** | 0.136ns |
| Interaction | 0.1093ns | 0.0122* | 0.0001*** | 0.000*** | 0.069ns |

*, **, and *** indicate significance at 0.05, 0.01, and 0.001 probability level.

Table S2 Analysis of variance (*P*-values) for the soil properties under two cropping systems and growth stages of sorghum, and yield traits under two cropping systems in 2022

| Source of variation | Soil total N content | Soil alkaline hydrolyzable N | Soil available P content | Soil available K content | Soil organic matter |
|---|---|---|---|---|---|
| Cropping system | 0.011* | 0.031* | 0.001*** | 0.045* | 0.016* |
| Growth stages | 0.036* | 0.002** | 0.056ns | 0.015** | 0.060ns |
| Interaction | 0.002** | 0.001*** | 0.071ns | 0.003** | 0.051ns |
| Source of variation | Panicle fresh weight | Panicle dry weight | Grain weight per panicle | Panicle fertility index | Grain yield |
| Cropping system | 0.027* | 0.026* | 0.011* | 0.050ns | 0.034* |

*, **, and *** indicate significance at 0.05, 0.01, and 0.001 probability level.
